# Central blood pressure and pulse wave velocity: relationship to target organ damage and cardiovascular morbidity-mortality in diabetic patients or metabolic syndrome. An observational prospective study. LOD-DIABETES study protocol

**DOI:** 10.1186/1471-2458-10-143

**Published:** 2010-03-18

**Authors:** Manuel A Gómez-Marcos, José I Recio-Rodríguez, Emiliano Rodríguez-Sánchez, Yolanda Castaño-Sánchez, Angela de Cabo-Laso, Benigna Sánchez-Salgado, Carmela Rodríguez-Martín, Carmen Castaño-Sánchez, Leticia Gómez-Sánchez, Luis García-Ortiz

**Affiliations:** 1 La Alamedilla Health Centre, Primary Care Research Unit, Castilla y León Health Service - SACYL, Salamanca, Spain

## Abstract

**Background:**

Diabetic patients show an increased prevalence of non-dipping arterial pressure pattern, target organ damage and elevated arterial stiffness. These alterations are associated with increased cardiovascular risk.

The objectives of this study are the following: to evaluate the prognostic value of central arterial pressure and pulse wave velocity in relation to the incidence and outcome of target organ damage and the appearance of cardiovascular episodes (cardiovascular mortality, myocardial infarction, chest pain and stroke) in patients with type 2 diabetes mellitus or metabolic syndrome.

**Methods/Design:**

**Design**: This is an observational prospective study with 5 years duration, of which the first year corresponds to patient inclusion and initial evaluation, and the remaining four years to follow-up.

**Setting**: The study will be carried out in the urban primary care setting.

**Study population**: Consecutive sampling will be used to include patients diagnosed with type 2 diabetes between 20-80 years of age. A total of 110 patients meeting all the inclusion criteria and none of the exclusion criteria will be included.

**Measurements**: Patient age and sex, family and personal history of cardiovascular disease, and cardiovascular risk factors. Height, weight, heart rate and abdominal circumference. Laboratory tests: hemoglobin, lipid profile, creatinine, microalbuminuria, glomerular filtration rate, blood glucose, glycosylated hemoglobin, blood insulin, fibrinogen and high sensitivity C-reactive protein. Clinical and 24-hour ambulatory (home) blood pressure monitoring and self-measured blood pressure. Common carotid artery ultrasound for the determination of mean carotid intima-media thickness. Electrocardiogram for assessing left ventricular hypertrophy. Ankle-brachial index. Retinal vascular study based on funduscopy with non-mydriatic retinography and evaluation of pulse wave morphology and pulse wave velocity using the SphygmoCor system. The medication used for diabetes, arterial hypertension and hyperlipidemia will be registered, together with antiplatelet drugs.

**Discussion:**

The results of this study will help to know and quantify the prognostic value of central arterial pressure and pulse wave velocity in relation to the evolution of the subclinical target organ damage markers and the possible incidence of cardiovascular events in patients with type 2 diabetes mellitus.

**Trial Registration:**

ClinicalTrials.gov Identifier: NCT01065155

## Background

The estimated prevalence of type 2 diabetes mellitus (DM2) in the industrialized world is 6-8% [1]. The prevalence of the disease is expected to increase in the coming years as a result of the increase in life expectancy in developed countries, and as a consequence of changes in life style in the developing world. It has been estimated that the number of diabetics will double over the next 25 years. As a result, by the year 2030, Spain can be expected to have over three million diabetic patients [1,2]. Cardiovascular diseases are the main causes of mortality among diabetic patients [3]. The incidence of coronary mortality in DM2 patients is four times greater than in the general population [4,5]. On the other hand, the prevalence of arterial hypertension (AHT) in DM2 is practically twice as high as in the non-diabetic population. Thus, 50-60% of all DM2 patients are hypertensive, and this percentage increases with age and the presence of nephropathy [6-8]. AHT in the diabetic patient is usually systolic [9-12], its control poses greater difficulties [13,14], and the nocturnal dip in pressure is smaller [15]. Moreover, the concomitant presence of both conditions (AHT and DM2) increases cardiovascular morbidity-mortality - with an increased incidence of coronary, cerebrovascular, and peripheral vascular disease, renal failure, heart failure and diabetic retinopathy, and a greater risk of death due to cardiovascular disease [16].

Clinical blood pressure (CBP) measurement remains the standard of reference, and has been a key element in predicting cardiovascular disease, but there is increasing evidence that home blood pressure (HBP) measurement by the patient, and particularly ambulatory blood pressure monitoring (ABPM), shows a stronger correlation to target organ damage and cardiovascular events [17-20]. The prognostic value of nocturnal blood pressure (BP), and particularly of the nocturnal decrease or dip in systolic blood pressure (SBP), seems to be greater than that of diurnal BP. Individuals with a lower nocturnal fall in BP have a greater prevalence of target organ damage and a less favorable outcome [21,22].

The presence of target organ damage increases the risk of clinical cardiovascular complications in DM2. In this context, left ventricular hypertrophy (LVH), assessed according to electrocardiographic criteria, increases the risk of coronary complications and stroke [23]. Silent worsening of renal function, reflected by increased creatinine levels, a drop in glomerular filtration rate (GFR), or an increase in protein excretion in urine, increase the risk of cardiovascular diseases [24,25]. Pathological ankle-brachial index (ABI) values show very good correlation to the development of coronary complications, the incidence of stroke, and cardiovascular mortality [26]. In addition, a number of prospective studies have shown carotid artery intima-media thickness (IMT) to be an independent risk factor for both coronary disease and stroke [27]. An increased IMT and/or the detection of atheroma plaques are associated with an up to four-fold increase in the relative risk of clinical complications of arteriosclerosis [28].

On the other hand, the evaluation of major artery stiffness is gaining importance as an indicator, since it is one of the main determining factors of the general condition of blood circulation [29,30]. Arterial stiffness is determined via two systems: pulse wave velocity (PWV)(time from arrival of the pulse wave to the carotid artery and femoral artery) and applanation radial tonometric pulse wave analysis for calculating aortic pressure, as well as the relationships between the latter and the peripheral pressure values [31-33]. Increased arterial stiffness or diminished arterial distensibility determined by pulse pressure implies increased cardiovascular risk. On the other hand, there is evidence that diabetes predisposes to premature vascular aging.

Considering the need in diabetic patients to better establish the prognostic value of central arterial pressure and pulse wave velocity in relation to the evolution of the target organ damage markers (left ventricle growth indicators, renal damage markers (microalbuminuria and diminished glomerular filtration), retinal damage and vascular markers (IMT of the carotid artery and ABI)) and the possible incidence of cardiovascular events in DM2 patients, an observational prospective study involving annual follow-up over at least four years is contemplated.

### The following objectives have been established

To evaluate the prognostic value of central arterial pressure and pulse wave velocity in relation to the incidence and outcome of renal (microalbuminuria, diminished glomerular filtration and the appearance of terminal renal failure), cardiac (LVH) and vascular damage (IMT and ABI), retinal vascular impairment, and the appearance of cardiovascular episodes (cardiovascular mortality, ischemic heart disease and stroke) in patients with DM2.

## Methods/Design

### Study design

An observational prospective study with a duration of 5 years, of which the first year corresponds to patient inclusion, and the remaining four years to follow-up. A minimum of 5 visits are contemplated, comprising a baseline visit at the time of patient inclusion in the study, and annual follow-up visits.

The study was approved by an independent ethics committee from the University Hospital of Salamanca (Spain), and all participants signed an informed consent document.

### Setting

The study will be carried out in the rural primary care setting, in the research unit of La Alamedilla primary care center, to which the general practitioners from two primary care centers serving a population of 46,000 inhabitants send patients for the evaluation of possible target organ damage and vascular risk.

### Study subjects

Consecutive inclusion will be made of the patients referred to the research unit and who meet the corresponding inclusion criteria, i.e., patients diagnosed with DM2 and aged 20-80 years, with none of the following exclusion criteria: patients unable to comply with the protocol requirements (psychological and/or cognitive disorders, failure to cooperate, educational limitations and problems for understanding written language, failure to sign the informed consent document), patients participating or who will participate in a clinical trial during the study, and patients with serious comorbidities representing a threat to life over the subsequent 12 months.

The sample size has been calculated to determine the time the patients take in suffering an event - the latter being interpreted as morbidity-mortality or the appearance of new target organ damage. To this effect, two groups will be considered, classified according to the median central arterial pressure value. The study accepts an alpha risk of 0.05 and a beta risk of 0.30 in two-sided contrasting for the detection of a minimum difference in relative risk between the two groups of ≥ 2.5 points, and considering an events rate within the high central arterial pressure group (values above the median) of 35% at the end of the study period. A total of 52 patients are required in each group, estimating a loss to follow-up of 10%. A final total of 110 patients therefore will be included.

### Measurements

#### Sociodemographic variables and cardiovascular risk factors

Patient age, sex, race, educational level and occupational status. Hypertension, dyslipidemia, alcohol consumption, smoking, physical activity and a history of premature cardiovascular disease (before 55 years of age in males and before 65 in females) in first-degree relatives.

#### Cardiovascular diseases

Coronary events: Myocardial infarction, unstable angina, stable angina, coronary revascularization. Cerebrovascular events: Ischemic stroke, intracranial hemorrhage, transient brain ischemia. Hospital admissions due to heart failure. Symptomatic peripheral arterial disease: Claudication over a distance of less than 100 m, resting pain, amputation. Heart failure. Atrial fibrillation.

#### Treatment variables

Treatment for diabetes. Antihypertensive treatment. Lipid-lowering treatment. Antiplatelet treatment.

#### Anthropometric measurements

Body weight is to be determined on two occasions using a homologated electronic scale (Seca 770) following due calibration (precision ± 0.1 kg), with the patient wearing light clothing and no shoes. These readings will be rounded to 100 g. Height in turn will be measured with a portable system (Seca 222), recording the average of two readings, and with the patient shoeless in the standing position. The values will be rounded to the closest centimeter. Body mass index (BMI)(kg/m^2^) will be calculated. Waist circumference will be measured using a flexible graduated measuring tape with the patient in the standing position without clothing. The upper border of the iliac crests are located, and the tape is wrapped around above this point, parallel to the floor, ensuring that it is adjusted without compressing the skin. The reading is taken at the end of a normal breath according to the recommendations of the 2007 SEEDO Conference [34].

#### Laboratory parameters

Lipids, creatinine, glucose, high-sensitivity C-reactive protein (CRP), fibrinogen and glycosylated hemoglobin (HbA1c) in blood and microalbuminuria. The parameters will be measured on a blind basis in the reference laboratory after patient fasting for at least 8 hours.

#### Office or clinical blood pressure

Office blood pressure measurement involves three measurements of systolic (SBP) and diastolic blood pressure (DBP), using the average of the last two, with a validated OMRON model M7 sphygmomanometer (Omron Health Care, Kyoto, Japan), by following the recommendations of the European Society of Hypertension [35]. Pulse pressure (PP) will be estimated with the mean values of the second and third measurements.

#### Home blood pressure (HBP)

This parameter will be measured using an OMROM model M7 sphygmomanometer (Omron Health Care, Kyoto, Japan). Three measurements will be made in the morning (between 6:00 and 9:00 a.m.), and three in the afternoon/evening (between 18:00 and 21:00 p.m.), over a period of 7 days, with a minimum interval of one minute between measurements, and excluding the first measurement in each case and the values corresponding to the first day of measurement [36]. The investigator will instruct the patients on how to perform the blood pressure recordings at home. Written instructions will be provided, as well as a self-registry sheet, to ensure correct pressure monitoring.

#### Ambulatory blood pressure monitoring (ABPM)

The ABPM was performed on a day of standard activity, with an adequate cuff for the size of the patient's arm. A control system, SpaceLabs 90207 model (Spacelabs Healthcare, Issaquah, Washington, USA), validated according to the protocol of the British Hypertension Society, was used [37]. The records in which the percentage of valid readings was ≥66% of the total and with valid readings at all times were considered to be valid. Furthermore, for the records to be evaluable, at least 14 measurements were required during the daytime period, or at least seven during the nightime or rest period. The monitor was scheduled for obtaining blood pressure measurements every 20 min during the daytime period and every 30 min during the rest period. The average and dispersión estimators of SBP and DBP were calculated during the 24-h, daytime and nightime periods, defined based on the diary reported by the patient. The patient completes a form specifying bedtime and wake-up time.

#### Assessment of carotid intima-media thickness

Carotid ultrasonography to assess IMT was performed by two investigators specifically trained for this before starting the study. A Sonosite Micromax ultrasound (Sonosite Inc., Bothell, Washington, USA) device paired with a 5-10 Mhz multifrequency high-resolution linear transducer with Sonocal software was used for performing automatic measurements of IMT for optimising reproducibility. Measurements were made of the primitive carotid after the examination of a longitudinal section of 10 mm at a distance of 1 cm from the bifurcation, performing measurements in the anterior or proximal wall, and in the posterior or distal wall in the lateral, anterior and posterior projections, following an axis perpendicular to the artery to discriminate two lines, one for the intima-blood interface and the other to the media-adventitious interface. A total of 6 measurements were obtained of the right carotid and other 6 of the left carotid, using average values (average IMT) and maximum values (maximum IMT) calculated by the software automatically. The measurements were obtained with the subject lying down, with the head extended and slightly turned opposite to the carotid examined, following the recommendations of the Manheim Carotid Intima-Media Thickness Consensus [38]. Finally, IMT image is frozen in telediastole by means of electrocardiogram triggering to avoid a confounding effect of pulsatile deformation of wall thickness and transferred to a computer. The average IMT was considered to be abnormal if it was above 0.9 mm, or if there were atherosclerotic plaques with a diameter over 1.5 mm, or a focal increase of 0.5 mm, or 50% of the adjacent IMT [16].

#### Evaluation of peripheral artery involvement

This was evaluated using the ankle-brachial index (ABI), performed in the morning without having consumed coffee or tobacco for at least 8 hours prior to measuring and an ambient temperature of 22-24°C. With the feet uncovered, in a supine decubitus position after 20 minutes of rest, the pressure in the lower extremities was measured using a portable Doppler system Minidop Es-100Vx (Hadeco, Inc. Arima, Miyamae-ku, Kawasaki, Japan) applying the probe at the anterior or posterior tibial artery at an angle of approximately 60° to the direction of blood flow. The transducer's cuff was quickly inflated in each ankle about 30 mmHg above the systolic pressure and the pressure was allowed to descend (by about 2 mmHg per second) until the first sound corresponding to the systolic pressure was heard. The blood pressure was also measured in both arms (measured twice at 3-5 minute intervals). The ABI was calculated separately for each foot by dividing the higher of the two systolic pressures in the ankle by the highest of the two systolic pressures in the arm [39]. It is considered subclinical organ damage if it is lower than 0.9 [16].

#### Cardiac assessment

The electrocardiographic examination was performed with a General Electric MAC 3.500 ECG System (Niskayuna, New York, USA), that measures automatically the voltage and duration of waves and estimates the criteria of the Cornell voltage-duration product (Cornell VDP) [40] to assess the LVH by the following equation: Men ((RaVL + SV3) * QRS) and women ((RaVL + SV3) * QRS + 6). LVH is defined as the voltage-duration product > 2,440 mm/ms (16). We will estimate left ventricular mass index (LVMI) by Novacode equation [41].

#### Renal assessment

The kidney damage was assessed by measuring creatinine plasma concentration, the glomerular filtration rate was estimated by CKD-EPI (Chronic Kidney Disease Epidemiology Collaboration) [42] and the MDRD-IDMS (Modification of Diet in Renal Disease-Isotopic Dilution Mass Spectrometry) [43] equation and proteinuria were assessed by the albumin/creatinine ratio following the 2007 European Society of Hypertension/European Society of Cardiology Guidelines criteria [16]. Subclinical organ damage was defined as plasma creatinine between 1.3 - 1.5 mg/dl in men and 1.2 - 1.4 mg/dl in women, glomerular filtration rate below 60 ml/min or albumin/creatinine ratio > 22 mg/gr in men and 31 mg/gr in women. Renal disease was defined as plasma creatinine of 1.5 mg/dl or higher in men and 1.4 mg/l in women or albumin/creatinine ratio > 300 mg/24 h.

#### Evaluation of retinal involvement

Retinography was performed with a Topcon TRC NW 200 non-mydriatic retinal camera (Topcon Europe B.C. Capelle a/d IJssel The Netherlands), obtaining images centered on the papilla. Once the images were captured, two independent observers classified them according to the Keith-Wagener classification for hypertensive retinopathy [44], with a third reading being performed in cases where there were discrepancies. Grades III or IV (hemorrhages or exudates, papillary edema) are considered to be associated with cardiovascular disease [16].

#### Evaluation of pulse wave velocity and central blood pressure

The pulse wave velocity (PWV) was calculated using the SphygmoCor System (Vx Pulse Wave Velocity), (AtCor Medical Pty Ltd Head Office, West Ryde, Australia) with the patient in the supine decubitus position. The carotid and femoral pulse wave was analyzed, estimating the delay in the electrocardiograma (ECG) wave and calculating the PWV. The space measurements were taken with a measuring tape from the suprasternal notch to the carotid and femoral arteries at the sensor location. A measurement of greater than 12 m/s is considered subclinical organ damage [16].

Using the SphygmoCor system (Px Pulse Wave Analysis)(Atcor Medical, Australia), with the patient in the sitting position and resting the arm on a rigid surface, pulse wave analysis will be made with a sensor in the radial artery - using mathematical transformation to estimate the aortic pulse wave. From the aortic wave morphology, central (aortic) arterial pressure will be estimated, along with central ventricular load, diastolic perfusion pressure, the subendocardial viability index, the pressure increment, central pulse pressure and the augmentation index (PWA), defined as the percentage increase in central pulse pressure: AIx = pressure increase/pulse pressure * 100 [33].

All explorations will be carried out on a yearly basis, and due registry will be made of any changes in the sociodemographic variables or cardiovascular risk factors, and of the appearance of events.

### Organization of data collection

The patients referred to the research unit of La Alamedilla primary care center will be interviewed by a contracted nurse specifically trained for the project. A history will be compiled and a basic exploration will be carried out. A 24-hour ambulatory blood pressure monitoring (ABPM) device will be fitted, and the patients will receive an OMRON M7 sphygmomanometer for self-measurement of blood pressure at home. Vascular exploration will be made (pulse analysis with the SphygmoCor system, ABI and ECG), and an appointment will be made for blood sampling. Posteriorly, carotid artery ultrasound will be performed by two specifically trained members of the research team. Data input will be made using the Teleform system (Autonomy Cardiff Vista, California, USA), with a questionnaire previously designed for the project, and exporting the data to the SPSS version 15.0 statistical package (SPSS Inc., Chicago, Illinois, USA) for posterior analysis.

### Statistics analysis

A first descriptive analysis will be made of the sociodemographic and clinical characteristics of the study groups. The data will be presented with the mean and 95% confidence interval in the case of quantitative variables, and as frequency distributions for qualitative variables. The Pearson chi-squared test will be used to analyze associations between qualitative variables. The Student t-test for independent samples will be used to compare the means for the two arterial pressure groups. In turn, the analysis of repeated measures will be based on the McNemar test for qualitative data and the Student t-test for paired data in application to quantitative data.

Survival will be analyzed with the Kaplan-Meier and actuarial methods on each visit and at the end of follow-up. The log-rank test will be used for comparing survival in the two groups. Lastly, a Cox regression model will be developed to estimate the event risk between the groups, adjusting for the possible confounding variables analyzed in the study.

Hypothesis contrasting will establish an alpha risk factor of 0.05 as the limit of statistical significance. The SPSS/PC+ version 15.0 statistical package will be used (SPSS Inc., Chicago, Illinois, USA).

### Quality control

Different processes will be carried out to guarantee study data quality and thus maximize the validity and reliability of the measurements of the results. To this effect, field work operative manuals have been developed, specifying the correct technique for performing each of the tests. Educative leaflets have also been developed to ensure that the patients correctly perform pressure measurement in the home - all these measures confirming the correct practical application of each technique. Meetings will be held on a weekly basis with the principal investigator of the study in order to analyze the entire process, and an annual report on the course of the study will be prepared.

### Ethical and legal issues

In order to guarantee data confidentiality, all the electronic and paper copies of the protocol, signed informed consent documents and results of the tests made in each of the patients will be kept locked in a safe place, and only the study investigators will have access to the data on the subjects who agree to participate in the study.

The study has been approved by the ethics committee and complies with Spanish data protection law 15/1999 and its recently developed specifications (Royal Decree (RD) 1720/2007). Knowledge and agreement to cooperate has been established with the implicated services, signed by the legal representative of the center.

## Discussion

The results of this study will be of great scientific relevance, since they will allow us to quantify the degree to which central arterial pressure and pulse wave velocity are related to the different target organ lesions (carotid IMT, peripheral arterial disease, kidney, retina and heart), and to analyze their course over time. To our knowledge, this is the first study to assess target organ damage in diabetic patients and its relationship to central arterial pressure and pulse wave velocity over a follow-up period of at least four years.

Pulse wave velocity is the gold standard for assessing the stiffness of the large arteries, and is an important predictor of cardiovascular events [45]. A recent review [46] has shown it to be correlated to patient age and arterial pressure, though its association to diabetes is not so clear. Discrepancies have been observed among different studies, and the recorded associations are weak [45]. Likewise, it is not certain that the different instruments used to assess arterial stiffness are equivalent. In this sense, a study in diabetic patients has concluded that pulse pressure and pulse wave velocity increase in diabetic individuals, though this is not associated to the augmentation index, and such parameters may not be reliable as a measure of arterial stiffness in diabetics [47]. In this same line, another study comparing different procedures for assessing arterial stiffness in diabetic subjects has concluded that further research is needed to clarify their usefulness in diabetic patients [48].

As regards the relationship between these arterial stiffness measures and target organ lesions and their course over time in diabetic individuals, the existing evidence is scarce and inconclusive. With the present study we hope to contribute evidence to clarify some of these issues.

### Study limitations

The main limitation of this study is its small sample size for assessing end events. However, this is remedied by the fact that the analysis will be made with the data of a multicenter study in which 800 patients will be included.

## Abbreviations

DM2: Diabetes mellitus 2; AH: Arterial hypertension; BMI: Body mass index; CBP: Clinical blood pressure; BP: Blood pressure; SBP: Systolic blood pressure; DPB: Diastolic blood pressure; HBP: Home blood pressure; ABPM: Ambulatory blood pressure monitoring; ABI: Ankle-brachial index; IMT: Intima-media thickness; HbA1c: Glycated hemoglobin; LVH: Left ventricular hypertrophy; PWV: Pulse wave velocity; ECG: Electrocardiographic; MDRD: Modification of Diet in Renal Disease.

## Competing interests

The authors declare that they have no competing interests.

## Authors' contributions

Conception of the idea for the study: MAGM and LGO. Development of the protocol, organization and funding: MAGM, LGO, ERS, JIRR, YCS, AdCL, BSS, CRM, CCS and LGS. Writing of the manuscript: MAGM. All the authors have read the draft critically, to make contributions, and have approved the final text.

## Pre-publication history

The pre-publication history for this paper can be accessed here:

http://www.biomedcentral.com/1471-2458/10/143/prepub
